# Development of a Web-Accessible Population Pharmacokinetic Service—Hemophilia (WAPPS-Hemo): Study Protocol

**DOI:** 10.2196/resprot.6558

**Published:** 2016-12-15

**Authors:** Alfonso Iorio, Arun Keepanasseril, Gary Foster, Tamara Navarro-Ruan, Alanna McEneny-King, Andrea N Edginton, Lehana Thabane

**Affiliations:** ^1^Health Information Research UnitDepartment of Clinical Epidemiology and BiostatisticsMcMaster UniversityHamilton, ONCanada; ^2^Hamilton Niagara Hemophilia ProgramDepartment of MedicineMcMaster UniversityHamilton, ONCanada; ^3^Department of Clinical Epidemiology and BiostatisticsMcMaster UniversityHamilton, ONCanada; ^4^Biostatistics UnitSt. Joseph’s HealthcareHamilton, ONCanada; ^5^School of PharmacyUniversity of WaterlooWaterloo, ONCanada

**Keywords:** hemophilia, population pharmacokinetics, factor VIII, factor IX, tailored prophylaxis

## Abstract

**Background:**

Individual pharmacokinetic assessment is a critical component of tailored prophylaxis for hemophilia patients. Population pharmacokinetics allows using individual sparse data, thus simplifying individual pharmacokinetic studies. Implementing population pharmacokinetics capacity for the hemophilia community is beyond individual reach and requires a system effort.

**Objective:**

The Web-Accessible Population Pharmacokinetic Service—Hemophilia (WAPPS-Hemo) project aims to assemble a database of patient pharmacokinetic data for all existing factor concentrates, develop and validate population pharmacokinetics models, and integrate these models within a Web-based calculator for individualized pharmacokinetic estimation in patients at participating treatment centers.

**Methods:**

Individual pharmacokinetic studies on factor VIII and IX concentrates will be sourced from pharmaceutical companies and independent investigators. All factor concentrate manufacturers, hemophilia treatment centers (HTCs), and independent investigators (identified via a systematic review of the literature) having on file pharmacokinetic data and willing to contribute full or sparse pharmacokinetic data will be eligible for participation. Multicompartmental modeling will be performed using a mixed-model approach for derivation and Bayesian forecasting for estimation of individual sparse data. NONMEM (ICON Development Solutions) will be used as modeling software.

**Results:**

The WAPPS-Hemo research network has been launched and is currently joined by 30 HTCs from across the world. We have gathered dense individual pharmacokinetic data on 878 subjects, including several replicates, on 21 different molecules from 17 different sources. We have collected sparse individual pharmacokinetic data on 289 subjects from the participating centers through the testing phase of the WAPPS-Hemo Web interface. We have developed prototypal population pharmacokinetics models for 11 molecules. The WAPPS-Hemo website (available at www.wapps-hemo.org, version 2.4), with core functionalities allowing hemophilia treaters to obtain individual pharmacokinetic estimates on sparse data points after 1 or more infusions of a factor concentrate, was launched for use within the research network in July 2015.

**Conclusions:**

The WAPPS-Hemo project and research network aims to make it easier to perform individual pharmacokinetic assessments on a reduced number of plasma samples by adoption of a population pharmacokinetics approach. The project will also gather data to substantially enhance the current knowledge about factor concentrate pharmacokinetics and sources of its variability in target populations.

**Trial Registration:**

ClinicalTrials.gov NCT02061072; https://clinicaltrials.gov/ct2/show/NCT02061072 (Archived by WebCite at http://www.webcitation.org/6mRK9bKP6)

## Introduction

Hemophilia A and B are X chromosome–linked bleeding disorders caused by mutations in the factor VIII (FVIII) and factor IX (FIX) genes. Both factors take part in the intrinsic pathway of blood coagulation. Affected individuals have severe, moderate, or mild forms of the diseases, defined by factor plasma levels of 1% or less, 2% to 5%, and 6% to 40%, respectively. Both hemophilia A and B are rare diseases; the prevalence of hemophilia A is 1 in 5000 male live births and that of hemophilia B is 1 in 30,000 [1,2]. Factor replacement therapy with plasma-derived or recombinant concentrates at regular intervals to prevent both bleeding and the resultant joint damage (ie, primary prophylaxis) is the mainstay of treatment of hemophilia [2,3].

Tailoring prophylaxis to individual patient characteristics has been suggested as an effective way to increase the net clinical benefit of hemophilia A and B treatment [4,5]. It has been demonstrated that hemophilia patients require either higher or lower prophylactic doses of clotting factor replacement products than the standard 20 to 50 IU/kg 2 to 3 times per week, and at least one-third of the patients can be effectively treated with lower doses or less frequent infusions [6-8]. A key element needed to tailor prophylaxis is the estimation of the pharmacokinetic disposition of FVIII/FIX at the individual level. The main barriers to pharmacokinetic assessment in clinical practice are the burden of multiple blood sampling required over 24 to 72 hours (usually 11 points for the classical approach) and the inconvenience for the clinician of performing the needed calculations, which, even adopting the approximation of a single compartment model kinetic, are beyond the expertise of most hemophilia treaters [9]. Furthermore, most of the newer extended half-life products require adoption of 2- or even 3- compartment models leading to substantially more complex calculations [10-13]. Population pharmacokinetics facilitates easier but still reliable estimation of individual parameters using reduced data points from each individual patient because it is based on mathematical models derived from previous knowledge from the entire available patient population. However, there are very limited readily accessible comprehensive hemophilia population pharmacokinetics applications available to clinicians. The Web-Accessible Population Pharmacokinetic Service—Hemophilia (WAPPS-Hemo) project aims to bridge this gap. The original study protocol was registered at ClinicalTrials.gov [NCT02061072].

The project has the following overarching objectives: (1) to empower hemophilia treatment by making it easier to perform individual pharmacokinetic assessments, (2) to allow for robust estimation of individual pharmacokinetic parameters with a reduced number of plasma samples, and (3) to enhance knowledge about the pharmacokinetics of FVIII and FIX.

The project has the following specific objectives: (1) to collect and compile published and unpublished individual classic pharmacokinetics data (individual patient data from independent investigators and factor concentrate manufacturers), (2) to create and make available population pharmacokinetic models for the concentrates derived from the data collected, (3) to develop a Web-based application intended to use the above models to calculate pharmacokinetic parameters for individual patients, (4) to test system functionality by use of fabricated test data, (5) to test the reliability of the pharmacokinetic reports provided by WAPPS-Hemo, and (6) to determine the potential value of this Web service for clinicians.

## Methods

### Study Design and Pharmacokinetics Repository

WAPPS-Hemo is a multicentric prospective project led by McMaster University, Hamilton, Ontario, Canada. The project is based on a population pharmacokinetics application hosted on a Web-accessible platform developed and run by the Health Information Research Unit, McMaster University. Pharmaceutical companies and independent investigators will be invited to provide already existing on-file individual pharmacokinetic data for developing the population pharmacokinetics models. All the factor concentrate manufacturers having on-file pharmacokinetic data and hemophilia treatment centers (HTCs) or independent investigators willing to contribute full or sparse pharmacokinetic data will be eligible for participation. All investigators of the pharmacokinetic studies identified via a systematic review of the literature will be contacted and invited to provide data.

### Research Network

Active hemophilia treaters will be invited to participate as coinvestigators and share ownership of the prospective pharmacokinetic database, to which they will be invited to provide plasma levels on sparse samples from eligible patients. They will be invited to provide feedback on the WAPPS-Hemo performance and functionality during the development phase of the project and to propose any substudy they could be interested to run in the network. Patient participation in the project will be mediated by their participating treatment center, as WAPPS-Hemo will not be directly available to patients. Initial emails will be sent out to the clinicians/hemophilia treaters to determine interest in this project. Once initial agreement of project participation has been confirmed, research agreements will be sent to facilitate data transfer. Research agreements will be operationalized offline and kept on file. No remuneration will be provided to either participating centers or patients.

### Anticipated Outcomes

The WAPPS-Hemo project will result in the availability of a repository of published and unpublished factor concentrate concentration-time datasets for hemophilia patients. Published data will be summarized in a systematic review and submitted for publication. Further, population pharmacokinetics models for the factor concentrates for which concentration-time data are available will be established. The model development process is described in a separate scientific report [14]. The extended version of this report will be available as part of the study documentation, while a synthetic version will be submitted for publication. A major outcome will also be that a Web-based WAPPS-Hemo engine will be made available to participating clinicians that will provide tailored pharmacokinetic estimates for their patients, including estimated terminal half-life; time to 1%, 2%, and 5% plasma clotting factor level; and expected plasma level of factor concentrate at 24, 48, and 72 hours postinfusion. The engine will also report the credibility estimates. Once the system is fully developed and validated we will consider releasing a version of the service available to clinicians unable or unwilling to participate in the research network and effort. However, we envision that any users, independent of the level of participation into the project, will require an identification and authentication process as well as the authorization to reuse anonymized data for modelling purposes. Indeed, quality and reliability of the data provided to the system and potentially used to refine the models require a mechanism to contact the user to perform data quality checks.

### Sample Size Considerations

We expect to be able to derive models for concentrates for which we have 20 or more preexisting densely sampled individual pharmacokinetic profiles. We expect to have data available for 4 or more different factor concentrates. We expect participation from 10 clinical centers with each center contributing data from approximately 10 patients.

### Project Development Phases

In order to fulfill the aims and objectives, the project will be operationalized as distinct but closely related work components as listed below. The work components will proceed in an iterant and parallel mode.

1. A systematic review to identify all existing publications reporting pharmacokinetic data on FVIII and FIX

2. Collection of individual pharmacokinetic data on file from investigators and factor concentrate manufacturers by signing bilateral data transfer agreements

3. Establishment of population pharmacokinetics models for factor concentrates

4. Creation of a Web-accessible platform, WAPPS-Hemo, to calculate patient-specific pharmacokinetic estimates from clinician-input concentration-time data

5. Integration of the population pharmacokinetics models into the WAPPS-Hemo Web service

6. Recruitment of HTCs and hemophilia treaters from across the world to the research network

7. User testing of the Web interface by participating centers by use of the Software Usability Score [15] and think-aloud techniques [16] using standardized fabricated patient data

8. Testing the reliability of the pharmacokinetic report provided by the WAPPS-Hemo Web service

### Development of the Web-Accessible Population Pharmacokinetic Service—Hemophilia Web Interface

#### Overview

The system will be developed by and hosted at the Health Information Research Unit, McMaster University, Ontario, Canada. A cluster of fully resilient Hewlett Packard servers (Windows Web servers in network load balancing configuration) for hosting the site, Microsoft SQL (Microsoft Corp) for the database, and a Windows server for the modelling software, located in 2 different buildings, will support the system platform. The system will incorporate fully mirrored hard disks and redundant https connection. All WAPPS-related URLs (eg, .com, .org, .ca) were blocked immediately at project funding notification. All the needed licenses will be acquired, including a single-site license for the population pharmacokinetics software, NONMEM (ICON Development Solutions). The website, database access interface, and back-end NONMEM interface will be programmed in Microsoft (Microsoft Corp) dot.Net programming language. The user interface will be device responsive.

#### System Development Approach and Architecture

**Figure 1:**
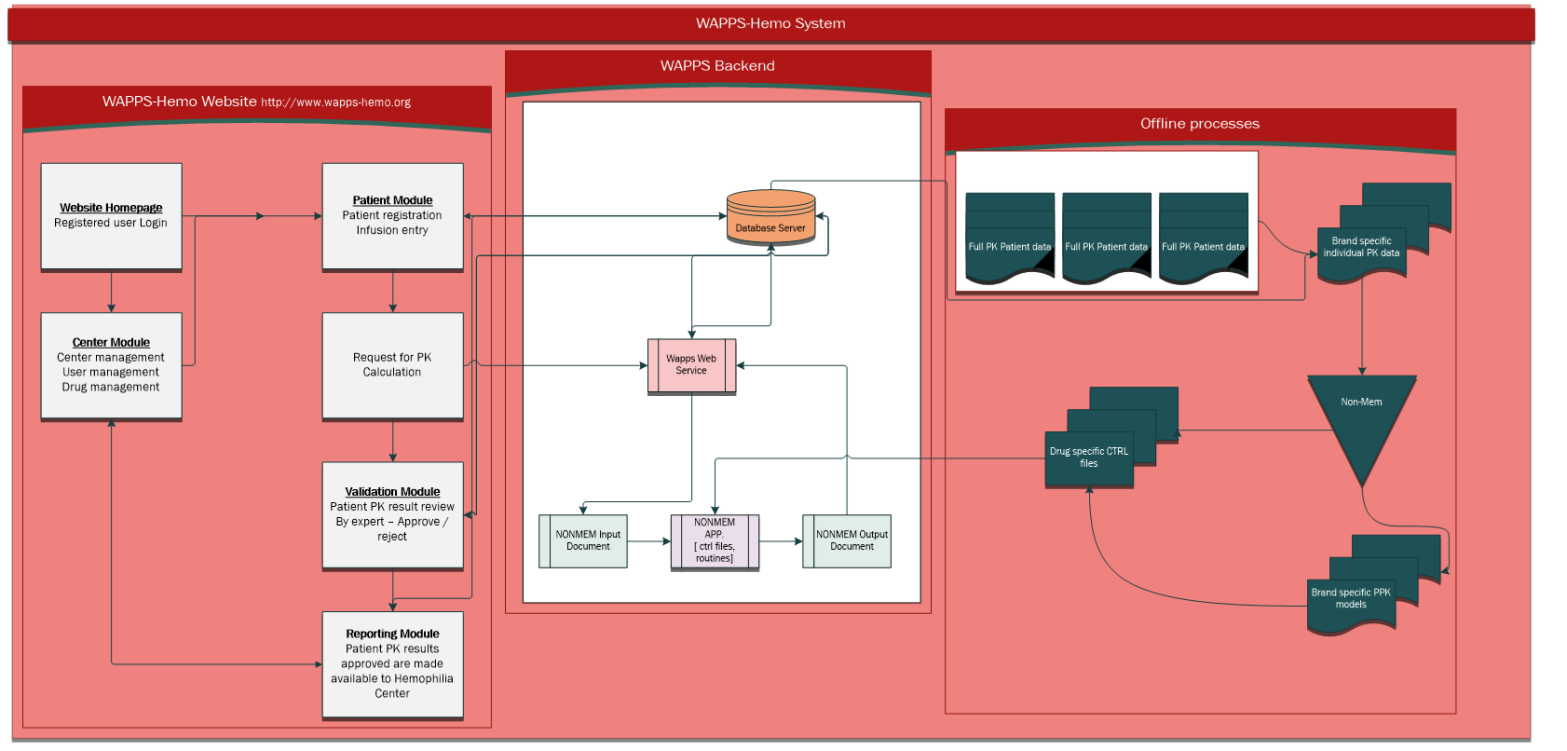
Schematic representation of the Web-Accessible Population Pharmacokinetic Service—Hemophilia (WAPPS-Hemo) system architecture. PK: pharmacokinetics; PPK: population pharmacokinetics; CTRL: control.

We will use Agile software developing methods to develop the WAPPS-Hemo. Agile methodology was selected over the conventional software development life cycle method because, unlike conventional methods, it allows the team to iteratively plan and strategize the development of the system [17]. We indeed postulated that the Agile strategy of developing the system in small iterations (known as sprints) is particularly suited to a project of dynamic nature like WAPPS-Hemo because it allows the team to adapt to changing requirements based on short-term goal-setting stemming from a large collaborating network of HTCs [18]. The system architecture schema is presented in Figure 1.

**Figure 2:**
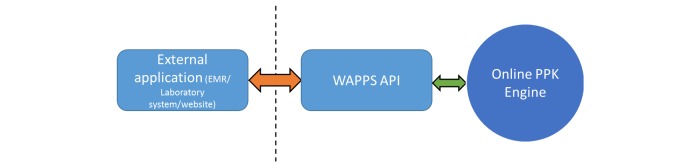
Schematic representation of the Web-Accessible Population Pharmacokinetic Service—Hemophilia (WAPPS-Hemo) application programming interface implementation. EMR: electronic medical record; API -application programming interface; PPK: population pharmacokinetics.

The system will be provided with an application program interface to facilitate potential integration with electronic medical record software, database, and clinical trial case report forms at a later stage (see Figure 2).

Field testing will be performed by each participating center. Initially, fabricated patient data will be used. Following successful registration, the centers will be provided with a standardized set of data and asked to generate records for those patients. Population pharmacokinetics estimates will be requested, reports generated, and feedback on the entire process gathered; we will troubleshoot any arising issues. We will conduct a formal usability evaluation using the Software Usability Scale, think-aloud technique, and focus group feedback to modify the service accordingly.

### Description of Expected Information Flow on the Website

#### User Registration

The home page of the website will have a section where a user can register or log in to the service. Registration will be open to participating centers/local investigators after they agree to and sign the research agreement. Registration requests will be validated before the center is activated. Data on the laboratory tests used by the center will be collected at the time of patient data input but will be stored as user characteristics for subsequent use for future patients from that center.

#### Patient Registration

**Figure 3:**
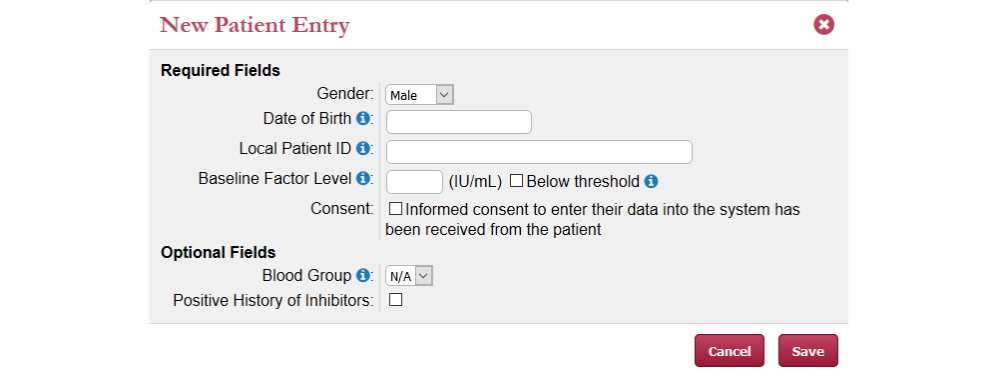
Web-Accessible Population Pharmacokinetic Service–Hemophilia new patient entry page.

The goal is to allow the participating center to identify their patients while still respecting privacy regulations. Patient identification is critical in allowing re-input of the same patient's data in the future and dispatching of the reports to the proper requester. The patient registration module will be activated for each participating center/investigator following ethics approval. The system will require a local patient identifier to be created for which any combination of alphanumeric codes will be accepted. Identifiable personal health information will be stored centrally, locally, or not stored at all in agreement with local privacy and ethical requirements. Patient date of birth, sex, diagnosis, and severity are the mandatory data required for patient registration. Optional fields may be collected as well, on each patient or prompted by specific characteristics (see Figure 3). We plan to include among optional fields blood group and inhibitors (actual and peak titer as Bethesda units/mL, date of peak, last measurement, and actual treatment regime). Ethnicity, race, genotype, or other information potentially associated with pharmacokinetics will or will not be added to the optional fields based on user feedback.

#### Infusion and Plasma Level Data Entry

**Figure 4:**
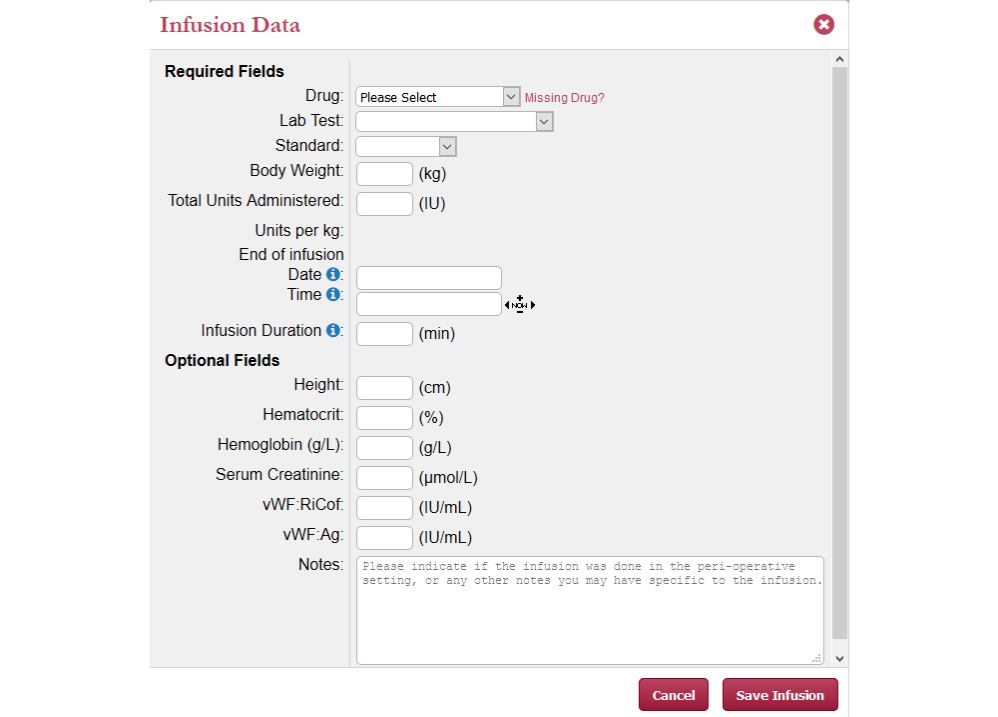
Web-Accessible Population Pharmacokinetic Service–Hemophilia measurement entry page.

After a patient record is created, an infusion record can be added (see Figure 4). This will require the input of patient body weight, the specific factor concentrate infused, the total dose infused, the infusion time and duration. Predose plasma factor level will be provided when available. Postinfusion factor levels will be input as sampling time and factor concentration in IU/mL. Samples for which the level falls below the detection threshold will be identified as below the level of quantitation.

#### Pharmacokinetic Estimation

After completion of the data input step, the user will request the estimation of the individual pharmacokinetic parameters. A congruency check will be performed on the submitted input data. Details about the development and evaluation of the underlying population pharmacokinetics models as well as details of the postmodelling Bayesian estimation are provided in a separate paper [14].

**Figure 5:**
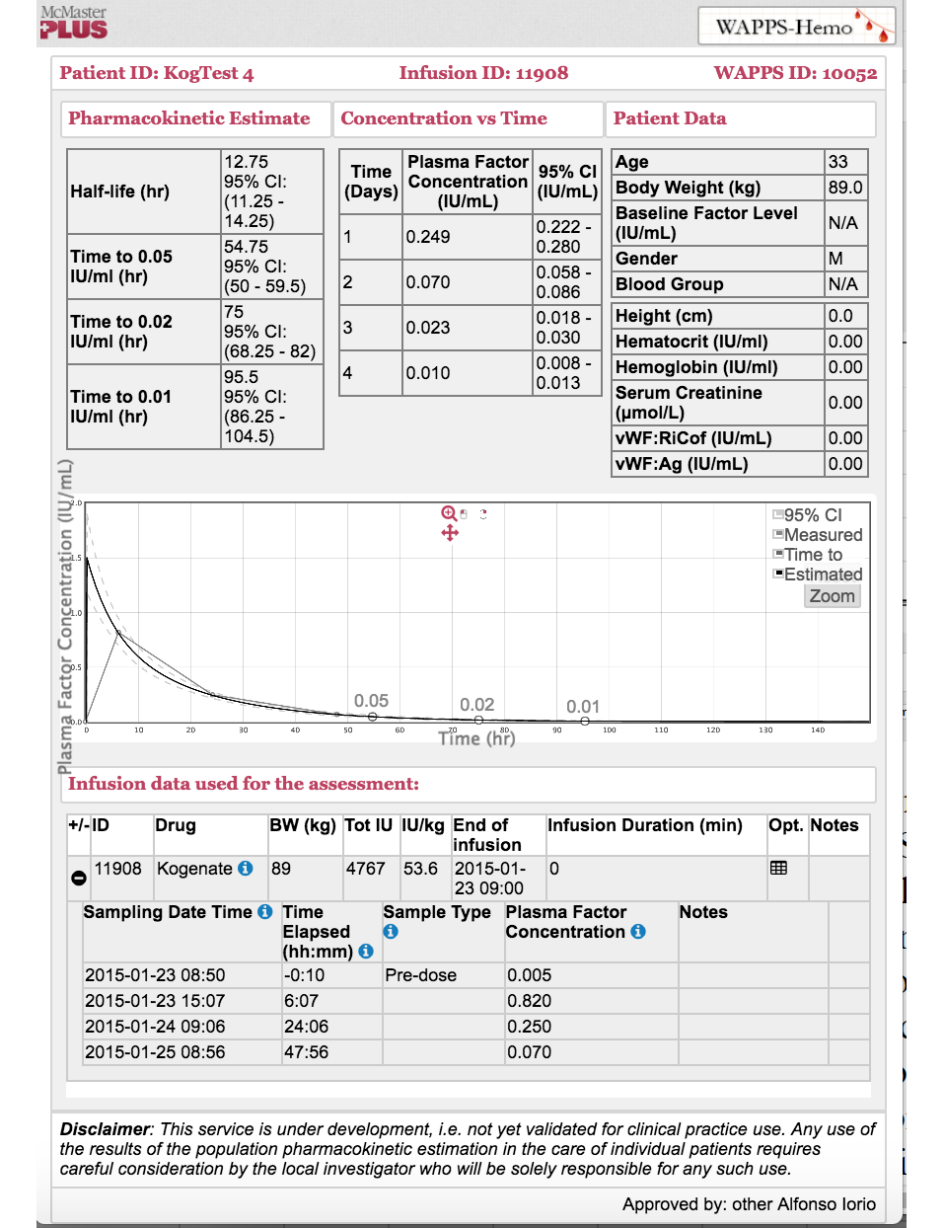
Web-Accessible Population Pharmacokinetic Service–Hemophilia result page.

### Reporting

Each individual infusion will be reported. The report will contain inputted data; terminal half-life; time to 0.05, 0.02, and 0.01 IU/mL of plasma factor level with Bayesian credibility intervals; expected plasma concentrations at 24, 48, and 72 hours; and an interactive graphical representation of the predicted postinfusion concentration-time profile (see Figure 5). To ensure that the data and estimates are reliably accurate, automated and human checks will be performed to determine congruency of the data and estimates before releasing the results. Therefore, there will be no real-time reporting of the data and pharmacokinetic estimates.

### Risks

There are no additional risks to the patient beyond the risk associated with standard of care at each participating center. During the initial development of the service we will not offer the Web application beyond the boundaries of the participating centers. The website will be made available to those in the research network during the testing phase using only fabricated data. Although participating centers will have the opportunity to input real patient data and receive pharmacokinetic reports, we will clearly identify the prototypal and experimental use of the system in both the research agreements and the website page as a “Research service not yet ready for clinical practice.” A clear disclaimer will indicate that “Any use of the results of the population pharmacokinetic estimates in the care of individual patients should not be considered part of the project in this phase.”

### Research Ethics Approval

The WAPPS-Hemo project has received ethical approval from the Hamilton Integrated Research Ethics Board. Only data already collected as part of other studies will be used for the establishment of population pharmacokinetics models and, as such, these studies have already received ethics approval. Legal approval for the use of the data will be sought from the owner of the data. Any other applicable procedures will also be followed.

The local investigator from the participating center will submit the WAPPS-Hemo study protocol, including a localized consent form, to their local ethical review board for approval. The coordinating center at McMaster will support the local investigator in the application to the local research ethics board if requested to do so. The local investigator will obtain informed consent for the input of patient data on the WAPPS-Hemo system from each participant whose data is provided. Copies of the ethical approval and informed consent letters will be stored locally. When a participating center submits patient data to WAPPS-Hemo, the investigator will need to declare that the patients have provided consent. Any important protocol modifications will be sent to the relevant parties through email.

### Data Access, Security, and Confidentiality

Only anonymized patient data already collected as part of clinical studies or on file from factor concentrate manufacturers will be collected for the development of models. No personal identifying information such as name or social insurance number will be collected.

In the prospective phase, users will be allowed to access and use the system only after a moderated registration process. The head of the participating center will be required to go through a registration process, and following validation by the core McMaster team, that person will receive credentials to access the website and will subsequently be able to authorize other users from the center to access the system. Each individual user will have unique access credentials and will be authorized to manage patients from the center she or he belongs to. Only authorized users will be able to create and access patient records and input the information required for the pharmacokinetic assessment.

For the initial identification of patients, the system will require a local patient identifier to be created for which any combination of alphanumeric codes will be accepted. It will then be the responsibility of the participating center to track the local patient WAPPS-Hemo identifier in the patient case report form. Each center will be free to adopt, in respect of their local ethical and privacy regulation and by-laws, any structured or unstructured way of identifying their patients, including the use of alphanumeric strings for patient first and last names. Whenever possible, use of an existing patient identifier from the local health care system as WAPPS identifier is recommended.

All data held at McMaster will be stored on a secure server with adequate physical, technical, and administrative safeguards. Physical access to the data center is limited to the information technology manager and programmers of the unit via a swipe card system and is monitored at all times by video surveillance. The administrative website and backup database will be accessible only over a virtual private network. The system will be available around the clock with expected downtime less than 0.1%. Access to the Web service will be tightly controlled via robust passwords. We will use a secure https connection for added security. Any data used for statistical analysis and reporting will have been previously anonymized. Only the research team will have access to the data.

Participants of this project will not be identified in any reports or publications. No paper records of the data will be maintained. Patients will have the right to request complete cancellation of their personal data through the local investigator or to completely deidentify any concentration-time points (this last information will be retained in the system if already used for scientific reporting for as long as allowed by current regulation).

### Results Dissemination Policy

As a part of a research project, results from all the above activities will be reported in peer-review publications. All scientific communications regarding the population pharmacokinetics models (whether abstract, scientific meeting, or peer-reviewed publication) stemming from this project will go through a formal approval step with all the entitled authors, including any factor concentrate manufacturer’s publication committee.

### Project Funding and Sustainability

The development and initial operation of the WAPPS-Hemo website is supported by a research grant, awarded as result of a competitive peer-reviewed grant competition by the Association of Hemophilia Center Directors of Canada Baxter Canadian Hemophilia Epidemiological Research Program. We plan to seek additional research funding and to sustain ongoing operation of the project with intramural funds from the Health Information Research Unit of McMaster University.

## Results

**Figure 6:**
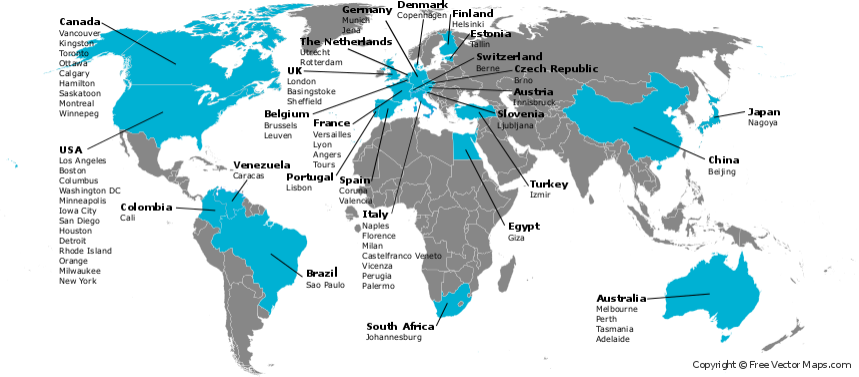
Web-Accessible Population Pharmacokinetic Service–Hemophilia research network.

The WAPPS-Hemo research network currently involves 47 registered active HTCs from across the world. Figure 6 illustrates the geographical distribution of the network.

An additional 26 HTCs are in various stages of the internal approval process, and 29 more HTCs have expressed interest in joining the network.

We have collected dense individual pharmacokinetic data on 878 subjects, including several replicates, on 21 different molecules from 17 different sources. We have collected sparse individual pharmacokinetic data on 289 subjects from the participating centers through the testing phase of the WAPPS-Hemo Web interface.

We have developed prototypal population pharmacokinetics models for 11 molecules. All developed models and methodological issues involved in the development and validation of models are reported in separate scientific reports [14,19]. In brief, assessment of validity of a system like WAPPS-Hemo is a complex process which will be covered in a stepwise fashion. After validation of the population models by bootstrapping and comparing population and structural estimates, we will prospectively validate the prediction by asking WAPPS-Hemo users to resample the patient after the pharmacokinetics has been estimated; the measurements will be used to assess the goodness (validity, precision) of the prediction. The final validation step will be to assess the impact of adopting WAPPS-Hemo–based forecasts to tailor treatment. This will require an ad hoc designed prospective clinical trial, currently in the planning stage.

The WAPPS-Hemo website (available at www.wapps-hemo.org, version 2.40), with core functionalities allowing hemophilia treaters to obtain individual pharmacokinetic estimates on sparse data points after 1 or more infusions of a factor concentrate, was launched for use within the research network. Asynchronous user support is currently made available via a monitored email service; we expect response time within the following working day, and a phone interaction can be started by the help desk when deemed to be necessary.

A formal usability study of the WAPPS-Hemo interface involving 13 participants (physicians, nurses, and clinical coordinators) from 2 centers in Canada, 1 in the United States, 1 in the United Kingdom, and 1 in Turkey has been performed. Two iterations resulted in new releases of the software interface. Detailed results will be reported in a separate publication. The WAPPS-Hemo interface has been translated into 11 languages: Arabic, Chinese, English, French, German, Italian, Japanese, Farsi, Portuguese, Spanish, and Turkish.

Two large prospective studies addressing the value of pharmacokinetic tailored prophylaxis, one in hemophilia A and one in hemophilia B, have been recently funded and are underway in the United States, both adopting WAPPS-Hemo as the population pharmacokinetics engine (personal communication).

## Discussion

### Overview

The WAPPS-Hemo service is the first dedicated population pharmacokinetics calculator available on an institutional website, essentially simplifying and facilitating individual pharmacokinetic assessment for treatment of hemophilia A and B. We expect that the widespread adoption of the system will lead to a reduction in the need for blood samples in individual patients, with particular benefit for the assessment of pharmacokinetics in pediatric patients. The capability of assessing pharmacokinetic parameters on sparse data will bear the potential to extend the use of pharmacokinetics to different scopes, including monitoring and optimizing prophylaxis regimes, identifying individual treatment targets, and switching from product to product. This should result in better care while optimizing resource utilization.

The WAPPS-Hemo system is built for progressive refinement of its own capabilities. Patient data will be periodically merged into the reference database of the system, and models will be periodically recalibrated. This continuous quality assurance process will enhance its capability to perform precise predictions.

The WAPPS-Hemo system is built to support research and knowledge translation. The system will progressively accrue individual pharmacokinetic data for both hemophilia A and B and different factor concentrates, which will be shared within the WAPPS-Hemo research network. The availability of a large database of pharmacokinetic data jointly with a research network of centers with a specific interest in pharmacokinetics will pave the way to a deeper understanding of the individual pharmacokinetic properties of different concentrates and will increase the evidence base used to treat hemophilia patients.

### Limitations

The main limitation of the current project design is the need for users to have some basic understanding of pharmacokinetics. Indeed, the system does not have built-in functions to support simulation of different dose regimens. Other limitations are integral to the need to demonstrate the true net impact of population pharmacokinetics modelling in hemophilia care, the identification of the most efficient sparse sampling protocols, and the identification of relevant covariates to maximize the impact of using a population pharmacokinetics approach.

### Conclusion

In summary, WAPPS-Hemo is well positioned and has become the largest pharmacokinetic data repository in the field. The interconnection of a Bayesian engine, population pharmacokinetic routines, and smart end-user interface constitute an innovative blend of different high-tech approaches with potential to impact the care of hemophilia.

## Abbreviations

FVIII: factor VIII
FIX: factor IX
HTC: hemophilia treatment center
WAPPS-Hemo: Web-Accessible Population Pharmacokinetic Service—Hemophilia

## References

[1] Stonebraker JS, Bolton-Maggs PH, Soucie JM, Walker I, Brooker M (2010). A study of variations in the reported haemophilia A prevalence around the world. Haemophilia.

[2] Stonebraker JS, Bolton-Maggs PH, Michael SJ, Walker I, Brooker M (2012). A study of variations in the reported haemophilia B prevalence around the world. Haemophilia.

[3] Iorio A, Marchesini E, Marcucci M, Stobart K, Chan AK (2011). Clotting factor concentrates given to prevent bleeding and bleeding-related complications in people with hemophilia A or B. Cochrane Database Syst Rev.

[4] Feldman BM, Pai M, Rivard GE, Israels S, Poon M, Demers C, Robinson S, Luke K, Wu JK, Gill K, Lillicrap D, Babyn P, McLimont M, Blanchette VS, Association of Hemophilia Clinic Directors of Canada Prophylaxis Study Group (2006). Tailored prophylaxis in severe hemophilia A: interim results from the first 5 years of the Canadian Hemophilia Primary Prophylaxis Study. J Thromb Haemost.

[5] Risebrough N, Oh P, Blanchette V, Curtin J, Hitzler J, Feldman BM (2008). Cost-utility analysis of Canadian tailored prophylaxis, primary prophylaxis and on-demand therapy in young children with severe haemophilia A. Haemophilia.

[6] Poonnoose P, Keshava S, Gibikote S, Feldman BM (2012). Outcome assessment and limitations. Haemophilia.

[7] Valentino LA, Mamonov V, Hellmann A, Quon DV, Chybicka A, Schroth P, Patrone L, Wong W (2012). A randomized comparison of two prophylaxis regimens and a paired comparison of on-demand and prophylaxis treatments in hemophilia A management. J Thromb Haemost.

[8] Howard TE, Yanover C, Mahlangu J, Krause A, Viel KR, Kasper CK, Pratt KP (2011). Haemophilia management: time to get personal?. Haemophilia.

[9] Björkman S, Carlsson M (1997). The pharmacokinetics of factor VIII and factor IX: methodology, pitfalls and applications. Haemophilia.

[10] Bulitta JB, Landersdorfer CB, Forrest A, Brown SV, Neely MN, Tsuji BT, Louie A (2011). Relevance of pharmacokinetic and pharmacodynamic modeling to clinical care of critically ill patients. Curr Pharm Biotechnol.

[11] Joerger M (2012). Covariate pharmacokinetic model building in oncology and its potential clinical relevance. AAPS J.

[12] Brekkan A, Berntorp E, Jensen K, Nielsen EI, Jönsson S (2016). Population pharmacokinetics of plasma-derived factor IX: procedures for dose individualization. J Thromb Haemost.

[13] Björkman S, Oh M, Spotts G, Schroth P, Fritsch S, Ewenstein BM, Casey K, Fischer K, Blanchette VS, Collins PW (2012). Population pharmacokinetics of recombinant factor VIII: the relationships of pharmacokinetics to age and body weight. Blood.

[14] McEnemy-King A, Foster G, Iorio A, Edginton A (2016). Data analysis plan for the development and evaluation of population pharmacokinetic models for incorporation into the Web-Accessible Population Pharmacokinetic Service–Hemophilia (WAPPS-Hemo) (forthcoming). J Med Internet Res.

[15] Brooke J, Jordan P, Thomas B, Weerdmeester B (1996). SUS: A quick and dirty usability scale. Usability Evaluation In Industry.

[16] Jaspers MW, Steen T, van den Bos C, Geenen M (2004). The think aloud method: a guide to user interface design. Int J Med Inform.

[17] Coram M, Bohner S (2005). The impact of agile methods on software project management. http://ieeexplore.ieee.org/xpls/abs_all.jsp?arnumber=1409937.

[18] Sadasivam RS, Delaughter K, Crenshaw K, Sobko HJ, Williams JH, Coley HL, Ray MN, Ford DE, Allison JJ, Houston TK (2011). Development of an interactive, Web-delivered system to increase provider-patient engagement in smoking cessation. J Med Internet Res.

[19] McEneny-King A, Iorio A, Foster G, Edginton AN (2016). The use of pharmacokinetics in dose individualization of factor VIII in the treatment of hemophilia A. Expert Opin Drug Metab Toxicol.

